# Heat-Related Mortality in India: Excess All-Cause Mortality Associated with the 2010 Ahmedabad Heat Wave

**DOI:** 10.1371/journal.pone.0091831

**Published:** 2014-03-14

**Authors:** Gulrez Shah Azhar, Dileep Mavalankar, Amruta Nori-Sarma, Ajit Rajiva, Priya Dutta, Anjali Jaiswal, Perry Sheffield, Kim Knowlton, Jeremy J. Hess, on behalf of the Ahmedabad HeatClimate Study Group, Gulrez Shah Azhar, Bhaskar Deol, Priya Shekhar Bhaskar, Jeremy Hess, Anjali Jaiswal, Radhika Khosla, Kim Knowlton, Mavalankar Mavalankar, Ajit Rajiva, Amruta Sarma, Perry Sheffield

**Affiliations:** IIPH-G; Bhaskar; IIPH-G; Emory University; NRDC; NRDC; NRDC and Mailman SPH, Columbia University; IIPH-G; IIPH-G; Fulbright Student Research Scholar; Icahn SOM at Mount Sinai; 1 Indian Institute of Public Health, Ahmedabad, Gujarat, India; 2 Public Health Foundation of India, New Delhi, India; 3 Columbia Mailman School of Public Health, New York, New York, United States of America; 4 Natural Resources Defense Council, New York, New York, United States of America; 5 Icahn School of Medicine at Mount Sinai, New York, New York, United States of America; 6 Department of Emergency Medicine, Emory University School of Medicine, Atlanta, Georgia, United States of America; 7 Department of Environmental Health, Emory University School of Public Health, Atlanta, Georgia, United States of America; Kagoshima University Graduate School of Medical and Dental Sciences, Japan

## Abstract

**Introduction:**

In the recent past, spells of extreme heat associated with appreciable mortality have been documented in developed countries, including North America and Europe. However, far fewer research reports are available from developing countries or specific cities in South Asia. In May 2010, Ahmedabad, India, faced a heat wave where the temperatures reached a high of 46.8°C with an apparent increase in mortality. The purpose of this study is to characterize the heat wave impact and assess the associated excess mortality.

**Methods:**

We conducted an analysis of all-cause mortality associated with a May 2010 heat wave in Ahmedabad, Gujarat, India, to determine whether extreme heat leads to excess mortality. Counts of all-cause deaths from May 1–31, 2010 were compared with the mean of counts from temporally matched periods in May 2009 and 2011 to calculate excess mortality. Other analyses included a 7-day moving average, mortality rate ratio analysis, and relationship between daily maximum temperature and daily all-cause death counts over the entire year of 2010, using month-wise correlations.

**Results:**

The May 2010 heat wave was associated with significant excess all-cause mortality. 4,462 all-cause deaths occurred, comprising an excess of 1,344 all-cause deaths, an estimated 43.1% increase when compared to the reference period (3,118 deaths). In monthly pair-wise comparisons for 2010, we found high correlations between mortality and daily maximum temperature during the locally hottest “summer” months of April (r = 0.69, p<0.001), May (r = 0.77, p<0.001), and June (r = 0.39, p<0.05). During a period of more intense heat (May 19–25, 2010), mortality rate ratios were 1.76 [95% CI 1.67–1.83, p<0.001] and 2.12 [95% CI 2.03–2.21] applying reference periods (May 12–18, 2010) from various years.

**Conclusion:**

The May 2010 heat wave in Ahmedabad, Gujarat, India had a substantial effect on all-cause excess mortality, even in this city where hot temperatures prevail through much of April-June.

## Introduction

Weather extremes can have significant public health impacts [1], [2], [3]. The global frequency of extreme weather events, including extreme precipitation, drought and resulting crop failure, tropical cyclones, and flooding has been increasing in recent years, consistent with anthropogenic climate change [4], [5]. These trends, which exhibit significant regional variability, are expected to continue in future as climate change becomes more pronounced [6]. A national assessment conducted by the Indian government on climate change projects increasing temperatures for India through the 21^st^ century, including increasing extreme heat events [7].

Temperature extremes are a major underlying weather-related cause of mortality in much of the world and the leading cause of directly-mediated weather-related mortality [8], [9], [10], [11], [12], [13]. Heat related morbidity and mortality can be due to either direct or indirect effects [14]. Direct effects include a spectrum of heat illness ranging from heat exhaustion to heat stroke; the indirect effects occur when heat exposure stresses underlying physiological systems and results in other specific manifestations such as renal insufficiency, acute cerebrovascular disease, and exacerbations of pulmonary disease [15].

A heat wave is a prolonged period of unusually and excessively hot weather, which may also be accompanied by high humidity. Definitions vary, in part because a heat wave is measured relative to the usual weather in the area and relative to normal temperatures for the season, and in part because there is no single best indicator from a public health perspective [12], [13] [16], [17]. The US National Oceanic & Atmospheric Administration (NOAA) defines a heat wave as a period of abnormally and uncomfortably hot and unusually humid weather lasting two or more days, and advisories are issued when these conditions are forecast.[18]. On a population basis, the impacts of high temperatures on mortality vary by location [19]. Heat wave conditions are known to amplify the effect of temperature on mortality [20] and several other effect modifiers have been identified [10], [21], [22]. According to the Indian Meteorology Department (IMD), a heat wave in India is declared when either there is an excess of 5°C over a normal daily historical maximum temperature (30 year average) of less than 40°C; or an excess of 4°C over a normal historical maximum temperature of more than 40°C. If the actual maximum temperature is above 45°C, is a heat wave is declared irrespective of the normal historical maximum temperature [18],[23].

Cities and urban areas experience higher levels of heat exposure than surrounding rural areas, due to the urban heat island effect whereby temperatures in urban areas are on average 3.5–12°C higher than those found outside city limits [24]. Similarly, urban microclimates have a role in creating higher urban temperatures in some parts of cities [25]. Urbanization can exacerbate heat exposures for residents of urban core areas, especially for developing countries where in-migration of rural poor and unplanned development of urban service systems may not be able to keep pace with demand. However, this on-going development also provides opportunities for municipalities to implement specific and targeted actions to mitigate the impacts of rising temperatures.

Among historic heat waves, some have been associated with large numbers of all-cause and cause-specific deaths, notably in France, Europe more broadly, Chicago and California [26], [27], [28], [29]. During the 2003 heat wave in France, excess mortality in 13 French cities varied from 4% to 142%, with a majority of cities experiencing excess mortality in the 20–50% range [26]. During the 2006 heat wave in California, there were an estimated 16,166 excess emergency department visits and 1,182 excess hospitalizations state-wide.[28]. In 2010, Moscow and Western Russia experienced a heat wave with ambient temperatures exceeding 39°C, and a high number of deaths, though specific estimates of excess morbidity and mortality have not been done [30].

India has had several historic heat waves. Most notably, in May 1998, India experienced a severe heat wave over a 2-week period, which was considered to be the worst in the previous 50 years [31]. The following year, a similar record-breaking event occurred in 1999 in north-west and central India. According to Kalsi et al (2001), during the summer of 1999, India experienced unprecedented heat in April, with maximum temperatures of 40°C or above for more than 14 days [32]. Another heat wave in 2003 caused more than 3,000 deaths in Andhra Pradesh [33].

In light of mounting epidemiologic evidence and the perception of a worsening threat to public health, several developed and even some developing countries have instituted prevention strategies and preparedness plans to minimize the human costs of increasing heat [34]. These include best practices for reducing municipal heat vulnerability and workplace heat-health promotion strategies, in cities from New York City to Abu Dhabi [35], [36], [37].

While the health impacts of heat waves, particularly impacts on mortality, have been explored for many regions of the world, there is relatively little information on specific impacts and characteristics of the relationship between excess heat exposure and health impacts in South Asia. We pursued the present analysis in an effort to begin filling this gap. Perhaps because extreme heat has not been recognized as a significant public health risk in India, or perhaps because the risk is now perceived to be increasing with climate change, heat health promotion strategies in India have only recently been made a matter of policy at the city government level. This study was done as part of a collaboration working to research and develop strategies for climate change adaptation in India. One of the first steps towards adaptation was to conduct the current analysis and risk assessment.

When considered with projected population densities, significant increases in premature heat-related mortality pose a threat to public health in India [38]. However, a discussion of the health effects of extreme heat is currently absent from India's national action plan for climate change [39].

In May 2010, Ahmedabad, India, a rapidly-growing city in the western state of Gujarat, experienced a heat wave. According to the India Meteorological Department criteria, the days of April 17–18 and May 13– 15, 17, and 20–25 in 2010, qualified as a “heat wave” with daily maximum temperatures varying between 44.5 – 46.8°C. There was an apparent increase in May 2010 all-cause mortality, though the true increase in mortality counts may have been under-reported [40], [41]. The objective of this ecological study is to characterize the heat wave's impacts on all-cause mortality and assess excess mortality associated with extreme heat exposure.

## Methods

To explore the relationship between the 2010 heat wave and mortality in Ahmedabad, we conducted an ecological analysis to evaluate potential relationships between daily all-cause mortality and maximum daily temperatures. We chose the May 2010 study period because of Ahmedabad's unprecedented heat wave, with maximum daily temperatures peaking at 46.8°C, a record high. The study was reviewed and approved by the Emory University Institutional Review Board and the Ethics committee at the Indian Institute of Public Health Gandhinagar.

Ahmedabad is the largest city in the state of Gujarat and the sixth largest city in the country, with an urbanized population of 5.571 million (2011) and with a metropolitan regional population of 6.35 million [42]. Extended population includes persons living outside of the Ahmedabad city limits but still within the municipal district of Ahmedabad. People living outside of the city limits are likely to have similar heat-related mortality risks as those in the urban core as these areas are also urbanized but not part of the municipality. The city's weather is usually dry and is hottest starting in March, with a seasonal monsoon rain period in July. The post-monsoon season is hot and wet and lasts until the end of October.

The Ahmedabad Municipal Corporation (AMC) is responsible for registering births and deaths in the city. We acquired anonymized and de-identified daily mortality data (day-wise death counts) from the AMC Office of the Registrar of Births and Deaths for the years 2009 to 2011. These deaths are inclusive of only those that occurred within city limits and not those in the extended city. The records for the daily mortality dataset were given to the authors under the necessary provision that the data is not distributed to the public and only the results obtained from the dataset may be used for publication purposes. Temperature data was obtained from the Indian Meteorology Department's Meteorological Aerodrome Report (METAR - a syntax used by the World Meteorological Organization or WMO), station at Ahmedabad airport (Station ID - VAAH, WMO ID 42647), located on the outskirts of the city, 8 Kilometres from the railway station.

### Estimating Excess All-Cause Mortality during the 2010 Heat Wave

Adapting the methodology of Anderson et al. [43], we applied 7-day moving averages for each day in May 2010 and compared them to averages from 2009 and 2011. For each day in May 2010, we compared the counts of all-cause deaths against a reference period comprised of the mean of all-cause death counts from corresponding days in May 2009 and May 2011. These years were chosen to control for population changes at the ecological level, since the population would be most similar in size and other demographic characteristics in the years immediately preceding and following the heat wave year. The daily number of excess all-cause deaths during May 2010 was calculated as the difference between the total monthly deaths in May 2010 minus the total reference period death counts for May from 2009 and 2011, again using an averaging method and a 7-day moving average method as described above. Percentage increases in May 2010 monthly excess mortality were also estimated, relative to the 2009 and 2011 reference period.

To estimate increases in mortality rate ratios (RR), applying the methodology of Lan et al. [44] and Rothman et al. [45], we considered the week of May 19–25, 2010, which included an acute 4-day extreme heat period from May 20–23, 2010 (as per the IMD's heat wave definition of daily maximum temperatures above 45 degrees Celsius) as the extreme heat wave period (H). The immediately preceding period, May 12–18, 2010 was considered as the reference period R1 and an alternative reference period R2 of May 19–25 from 2009 and 2011. RR were calculated for both reference periods R1 and R2. We assumed that the population size changed little over this period, cancelling the person-time units from the numerator and denominator. Thus, the simplified rate ratio “RR” was calculated using the formula RR  =  H/R. The 95% confidence intervals (CI) for RR were calculated as exp (ln (RR) ± 1.96(H^−1^+R^−1^)∧0.5). To minimize the heat's potential effects on all-cause mortality in the reference period, we also used an alternative reference period to calculate RRs, applying averaged all-cause mortality from May 19–25, 2009 and 2011 to re-estimate RRs.

Monthly correlations in 2010 for monthly maximum temperature and total monthly counts of premature all-cause mortality were also estimated.

Statistical analysis software SPSS 20 and Microsoft Excel (2013) was used for all analyses including generation of descriptive statistics, moving averages, and mortality rate ratios. Statistical significance of differences in mean values (temperatures and mortality) were calculated using two-tailed paired students T-tests.

## Results

**Figure 1:**
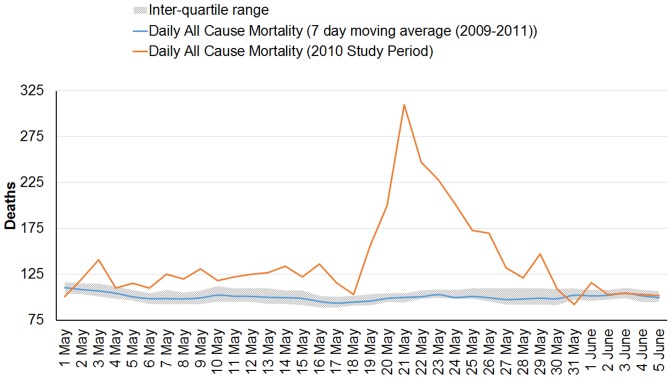
Daily mortality counts in May 2010 heat wave, versus corresponding days in 2009 and 2011. Daily mortality counts during the Ahmedabad, India May 2010 heat wave study period (in red), compared with average May all-cause mortality rates. Shown for comparison as a reference period are the mean (blue line) and interquartile range (hatched area) of a 7-day moving average of mortality in May of the preceding (2009) and following (2011) years.

The average daily mortality during the extreme heat wave period (as shown in Table 1) was estimated at 143.9 ±48.13, significantly higher than the average of 100.6 ±10.34 for the reference period (p<0.001 for the difference). This yields an estimated excess mortality in May 2010 of 1,344 deaths from May 1–31, 2010 an increase of 43.1% above the reference period. Other analytical approaches yielded similar results: comparison of daily counts for the extreme heat wave period compared with daily averages from the reference period yielded an excess of 1,353.5 deaths (a 42.88% increase), while a comparison using the 7-day moving average method yielded 1,334.9 deaths (a 42.81% increase). All results are consistent and suggest slightly over 43% excess deaths during May 2010. Figure 1 illustrates the daily mortality counts in May 2010 heat wave, versus corresponding days in 2009 and 2011.

**Table 1:** Daily temperatures and all-cause deaths for the month of May in Ahmedabad, Gujarat, India.

	Standard analysis			7-day moving average analysis	
	2010	Average for 2009 and 2011	Excess in 2010 (%)	Average for 2009 and 2011	Excess in 2010 (%)
Total Deaths	4462	3118	1344 (43.10)	3120.07	1334.93 (42.81)
Average daily Mortalities (deaths / day)	143.94±48.13	100.58±10.34	43.36 (43.11)	100.65±2.66	43.29 (43.01)
Maximum Temperature (°C)	42.81±1.25	41.05±1.27	1.76 (4.28)		
Minimum Temperature (°C)	28.45±1.50	28.25±1.41	0.19 (0.68)		
Mean Temperature (°C)	35.65±1.33	34.39±1.21	1.26 (3.66)		

Using mortality RR calculations, the RR is 1.76 [95% CI 1.67–1.83] (p<0.05) compared to a reference period (R1) in the preceding week (May 12–18, 2010). Applying the alternative reference period of May 19–25 from 2009 and 2011 (R2), the RR was significantly higher at 2.12 [95% CI 2.03–2.21] (p<0.001).

Table 2 shows correlations to quantify the strength of observed relationships between temperature and mortality. We found moderate to high correlations between monthly total all-cause deaths and monthly maximum temperature in the summer months while the same were negative for winter months. For May 2010, when the heat wave was recorded in Ahmedabad, the correlation was the highest (r = 0.775; p<0.001). The gender distribution as shown in Table 3 highlights significantly more female deaths in the summer months and the heat wave period.

**Table 2:** Month-wise correlations between monthly maximum temperature (°C) and total monthly all-cause mortality counts, 2010.

	January	February	March	April	May	June	July	August	September	October	November	December
Pearson Correlation Coefficient	−0.028^#^	−0.160^#^	0.360^*^	0.701^**^	0.775^**^	0.393^*^	0.503^**^	0.414^*^	0.083^#^	0.477^**^	−0.371^*^	0.290^#^

Note: *p<0.05, **p<0.01, ^#^non-significant.

**Table 3:** Gender distribution of decedents during the Heat Wave and the reference periods.

		Men	Women	Total
	Total (2009–2011)	68977	47021	115998
	Average deaths/day (2009 & 2011)	62.73	42.14	104.87
2010	Average deaths per day	63.52	44.55	108.07
	Excess deaths	287.50	881.00	1168.50
	Average excess deaths per day	0.79	2.41	3.20
May 2010	Deaths	2462	2000	4462
	Excess deaths	639	705	1344
	Average Excess deaths per day	20.61	22.74	43.35
	Average deaths excluding May 2010	62.04	42.69	104.74
Heat Wave Period (19–25th May2010)	Deaths	791	724	1515
	Excess deaths	373.50	427	800.50
	Average excess deaths per day	53.36	61	114.36
	Ratio (19–25^th^ May 2010)	1.89	2.44	2.12
P value Compared with 2009 & 2011	p for 19–25th May 2010	0.002521923	0.000647	0.000887
	p for May 2010	6.35662E-05	3.11E-05	2.4E-05
	p for entire year	0.332967427	0.000688	0.018211

For 2010, yearly mean maximum temperature was 33.9±5.0°C. This is significantly lower than the yearly average maximum temperature for 2009 and 2011, which was 35±4.8°C (p<0.001). For May 2010, the mean maximum temperature was 42.8±1.3°C. This is significantly higher than the May average maximum temperature for 2009 and 2011 which was 41.0±0.7°C (p<0.001) (Table 1). Similarly, the 2010 yearly mean and median daily mortality were 108±23 and 106 [IQR = 24] respectively and the May mean and median mortality were 143±48 and 125 respectively.

**Figure 2:**
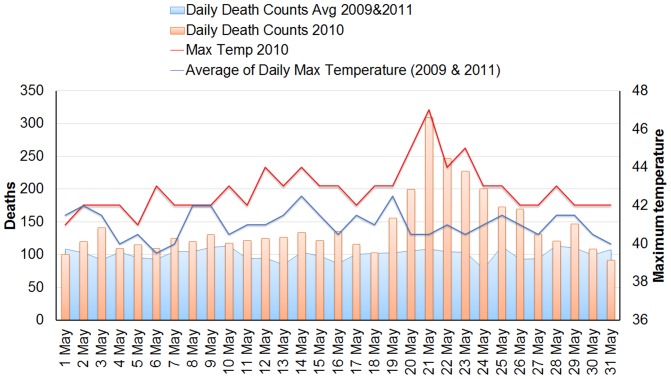
Temperatures and daily all-cause mortality, 2010 study period vs. 2009–2011 reference period. Daily maximum temperature versus daily all-cause mortality for the study period (1–31 May 2010), and the reference period (mean of corresponding values from days in May 2009 and 2011.

As illustrated in Figure 1, in May 2010 the number of daily deaths (though higher than average) increased greatly on May 18, 2010, reached the highest level on May 21, 2010, and returned to a more typical range on May 28, 2010. This pattern corresponds to increases in daily maximum temperature that occur during the May 2010 heat wave period, with an apparent increased association when the temperature crosses a threshold (43 °C for maximum or 36°C for mean temperature). There may be some mortality displacement on May 31, 2010 at which time daily mean mortality fell below the average, perhaps in short-term response to the large numbers of heat-vulnerable who had perished in prior days. Figure 2 shows the time course of both temperatures and death counts in both the 2010 study period and the reference period.

## Discussion

Although similar heat-mortality studies have been conducted in several European, American and Chinese cities, this is the first time that this relationship has been documented for any city in India. The findings are consistent with prior work that shows heat wave periods associated with overall excess all-cause mortality. Using a smoothed 7-day average calculation as well as a rough average estimate to calculate baseline measures, we estimate that the mortalities occurring during the May 2010 heat wave represent a 43% increase in mortality when compared with the same time period of other years in Ahmedabad. This amounts to an additional 1,350 deaths in the city during the heat wave period.

There was a similarly elevated mortality rate ratio of 1.76 [1.67–1.83 95% CI] during the shorter extreme heat wave period H from May 19–25, 2010. This estimate is considerably higher than one for China's 2010 heat wave [46] of 1.41 [95% CI 1.22–1.63].

One issue with our approach to the RR calculation is that it may underestimate the effects of extreme heat exposure as temperatures were already elevated in the May 12–18 reference period. To minimize the potential impact of the abnormally warm May 2010 temperatures on estimates of risk for all-cause mortality in the reference period, we applied an alternative non-2010 reference period to calculate RRs, and found a significant increase in the RR of 2.12 (95% CI 2.03–2.21; p<0.001).

Using historical exposure-response functions for temperature-heat relationships and various approaches to incorporating future adaptation activities, several projections of climate change impacts on heat mortality in various settings around the world have projected significantly increased risk in coming decades. These studies show an almost two-fold increase in the projected mortalities[47]. Our findings suggest that there is likely to be concern for similarly increased risk in the Ahmedabad region.

We observed (Figure 2) that the increase in temperatures and mortalities were concurrent, *i.e.*, there was no apparent lag time from the increase in temperatures to the increase in mortalities for the heat wave period in May 2010. Future time series analyses can investigate lag structures more fully, perhaps comparing mortality temperature lag times between tropical and temperate regions.

The analysis has several limitations. First, while the outcome data are generally reliable, there are several potential issues with mortality data in India. Reporting conventions are not uniform across the country. Even in one location reporting conventions are not always adhered to uniformly across time. In some settings some causes of deaths, though not the deaths themselves, are not accurately reported if they are considered sensitive. This has the potential to bias our results, likely toward the null, as it could lead to an understatement of the mortality risk during a heat wave period if not all of the deaths during that period were accounted for in the data set at a differential rate compared with the baseline. Assuming that reporting conventions remained stable during the study period, however, these issues should not affect our results, but they may be important if our results are compared with those from other areas in India.

A related concern is that not all deaths are reported. In the urban region of Ahmedabad, deaths taking place at home are likely to have been missed, and deaths among the homeless are unlikely to have been registered, potentially resulting in an underestimation of the effect if a greater proportion of deaths occurred at home or among the homeless during the heat wave period. This reporting bias may also have served as a confounder if there was increased likelihood of heat exposure and death and decreased likelihood of death reporting for a particular population, *e.g*., daily laborers, during the exposure period. Based on the data available, we were not able to control for these potential effects.

Second, the outcome is all-cause mortality, and as such our estimate may be an overestimate of the true effect of the heat wave on mortality as we were not able to perform a cause-specific analysis and isolate heat-related deaths. As we compared all-cause mortality in 2010 with a running average derived from seasonally similar time periods, however, this bias is likely to be relatively small as using the Integrated Disease Surveillance Project (IDSP) data (the disease surveillance system in India) during April to June 2010 we ruled out any major disease outbreaks for that period. Moreover, risks for cause-specific mortality would have been interesting to compare if the causes of death were available. Evaluations of whether a lagged effect of extreme heat on mortality exists in Ahmedabad could be a part of future studies that involve time-series analysis.

Third, because we are not able to stratify the deaths by demographics (apart from gender, where differences were insignificant) or socioeconomic status, our findings provide little specific insight into factors that may affect risk. Similarly, data disaggregated on socio-economic and socio-demographic variables would have provided useful insights on vulnerability. More detailed investigation and documentation of individual cases with cause of death and linkage of location and demographic and other factors will be needed to conduct a more nuanced evaluation of specific risk factors.

Fourth, a further complication exists in the placement of the temperature gauge that has been used historically to collect data in Ahmedabad. The daily maximum temperatures recorded within the city where residents are exposed to extreme heat may be higher than daily reports, as the current local source of daily temperature data is from an IMD monitoring station on the outskirts of the city. The degree of Ahmedabad's urban heat island effect is starting to be evaluated and may offer opportunities to fine-tune local temperature reporting. Future work that considers possible alternatives to the definition of a heat wave, with more health-relevant measures included, are areas of on-going research for the Ahmedabad Heat and Climate Study Group.

Fifth, for those persons who were indoors, these measures of ambient temperature are proxies of indoor conditions. Indoor residential temperatures would depend on a number of built environment factors including building materials, ventilation, nearby vegetation, and additional heat sources and sinks. A survey of relative measures of indoor versus outdoor temperatures across Ahmedabad was beyond the scope of this study. It is unknown whether ambient daily temperature measures are an under- or over-estimate of indoor room temperature for those individuals who died indoors, as neither specific information regarding the location of the decedents nor indoor exposure information is available

Finally, correlation in an ecological analysis alone does not indicate causation. There might be some role of confounding variables, such as air pollution, and / or other sources of bias. Air pollution effects were not the focus of this descriptive study of the May 2010 heat wave's effect on excess mortality in Ahmedabad. There could be possible interactive effects of heat and particulate or ozone air pollution, which would be a promising area for future study. The authors do not have access to the archived 2010 daily ozone or particulate air pollution data for the study period, though this data might help establish typical concentrations for comparison with the heat wave period. For particulate concentrations, Guttikunda and Jawahar (2012)[48] show monthly average PM10 concentrations from Ahmedabad for 2009–2010. May 2010 showed the highest monthly values for PM10 concentrations, but not to an extent that is likely to account for the dramatic increase in mortality that Ahmedabad experienced in May 2010. Moreover, prior studies including Anderson and Bell (2009)[49] and Hajat et al. (2006)[20] support the case that heat has a substantial, independent effect on mortality, which is independent of air pollution. Other potential confounders that were considered include infectious disease outbreaks (none were reported for the state of Gujarat in the weeks 2010 around the May heat wave), holidays, and other administrative issues that might have affected reporting.

Despite these potential concerns, the results of this study pose interesting questions and make findings regarding health protection against extreme heat in the region. This study is innovative and provides valuable data analysis since it is the first to examine the effects of extreme heat in a developing country setting. Such studies and findings are absent from the literature, hampering efforts to protect public health and to project the potential health impacts of climate change in developing countries.

One key question relates to the optimal definitions of extreme heat from the public health perspective and for early warning systems. This study evaluated mortality rate ratios during time periods that fell both within and outside the currently operational definitions of a “heat wave” and “extreme heat days” according to the Indian Meteorology Department, as described in the Introduction. Our findings suggest that the IMD definitions may underestimate the impacts of extreme heat on health because under the current systems IMD threshold does not formally account for public health effects of extreme heat. For public health purposes the IMD definitions may not be as useful as a definition with lower thresholds that are observed to correlate with public health outcomes more directly. Short and medium-range temperature forecasts, along with appropriate thresholds based on observed population health effects, could be used to generate early warnings of extreme heat that might significantly reduce the number of heat-related deaths.

To be most effective, as examined as part of this study, early warning systems also require measures to build preparedness and response capacity for medical and public health professionals and improved coordination and sensitization of staff working across city government and the medical facilities. Public awareness about the harmful impact of exposure to heat on human health is also critical to saving lives. Public awareness messages must be simple and easy to understand. They may include suggestions to check weather forecasts, stay in the shade, maintain hydration, wear appropriate clothing, avoid physical activity in the hottest part of the day, and check on neighbors and vulnerable members of the community.

In addition to early warning systems and preparedness plans, partnerships and information-sharing between various government agencies and key stakeholders needs to be developed and nurtured to facilitate the interdisciplinary work and effective implementation. Interventions also must be coupled with evaluation plans to determine their efficacy in the local context. Information and data-sharing channels have to be kept open and working to facilitate operations and evaluation activities. Appropriate health system improvements have to be made to ensure adequate response to health emergencies. International partnerships can also provide a strong base for knowledge-sharing and technical support, as evidenced by this collaborative study.

## Conclusions

In May 2010, the city of Ahmedabad in the state of Gujarat, India, experienced a heat wave with record-breaking maximum temperatures. During this heat wave an estimated excess 1,344 deaths occurred relative to a combined May 2009 and May 2011 reference period. This finding is consistent across several methods used for gauging the extra deaths. The May 2010 heat wave period represent a 43% increase in mortality when compared with the same time period of 2009 and 2011. The RR of an “extreme heat period” from 19–25 May was 1.76 (95% CI 1.67–1.83, p<0.001). Correlation coefficients between monthly maximum temperatures and monthly mortality show a significant relationship for the months of April (r = 0.701, p<0.001), May (r = 0.775, p<0.001). This paper aims to draw attention to extreme heat as a relatively under-appreciated public health risk in India, and provide some insight into the temperatures at which this risk appears to be marked on a population level. Given the trends associated with climate change, dangerous periods of extreme heat are likely to occur more frequently, suggesting the need for measures to reduce population vulnerability currently and in future. Heat wave-related mortality merits further analysis in order to reduce harmful health effects among India's most vulnerable and to help India adapt to the effects of climate change by increasing resilience to extreme heat.
