# Correction: Heat-Related Mortality in India: Excess All-Cause Mortality Associated with the 2010 Ahmedabad Heat Wave

**DOI:** 10.1371/journal.pone.0109457

**Published:** 2014-09-25

**Authors:** The PLOS ONE Staff

Natural Resources Defense Council was omitted as a funder for this study. Please refer to the correct funding statement below:

Dr. Hess's work on this project was via a consultancy arrangement with the Natural Resources Defense Council (NRDC) funded by the Climate and Knowledge Development Network (CDKN). This work was funded by a grant from the Climate Knowledge Development Network. The funders had no role in study design, data collection and analysis, decision to publish, or preparation of the manuscript.
